# Prevalence and Morphological Classification of Persistent Metopic Suture in Adult Autopsy Cases: A Forensic Anatomical Study from Western Türkiye

**DOI:** 10.3390/diagnostics16030415

**Published:** 2026-01-30

**Authors:** Gökmen Karabağ, Volkan Zeybek, Ahmet Küpeli, Mehmet Sunay Yavuz, Mahmut Aşırdizer, Ertuğrul Tatlısumak, Aslıhan Teyin

**Affiliations:** 1 Department of Forensic Medicine, Faculty of Medicine, Manisa Celal Bayar University, 45030 Manisa, Turkey; drvolkanzeybek@gmail.com (V.Z.); sunayyavuz@yahoo.com (M.S.Y.); 2 Manisa Forensic Medicine Department, Council of Forensic Medicine, 45020 Manisa, Turkey; drakupeli@yahoo.com; 3 Department of Forensic Medicine, Faculty of Medicine, Bahçeşehir University, 34480 İstanbul, Turkey; mahmut.asirdizer@bau.edu.tr; 4 Department of Anatomy, Faculty of Medicine, Manisa Celal Bayar University, 45030 Manisa, Turkey; ertugrul.tatlisumak@cbu.edu.tr; 5 İzmir Forensic Medicine Department, Council of Forensic Medicine, 35530 Manisa, Turkey; aslihanteyin@hotmail.com

**Keywords:** metopic suture, metopism, forensic autopsy, cranial variation, anatomical variant, persistent frontal suture

## Abstract

**Background:** Persistent metopic suture represents a normal anatomical variant that may persist into adulthood and can be misinterpreted as a frontal skull fracture, particularly in trauma-related forensic cases. Despite its clinical and medico-legal relevance, data derived from autopsy-based evaluations remain limited, with most prevalence studies relying on dry skull collections or radiological series. This study aimed to determine the prevalence and morphological characteristics of persistent metopic suture in adult autopsy cases and to evaluate its distribution according to age, sex, and cause of death. **Methods:** This cross-sectional study included 500 consecutive adult autopsy cases (≥18 years). The frontal bone was directly inspected during autopsy for the presence of metopic suture, which was classified as complete or incomplete. Descriptive statistics were applied, and associations between metopism and sex, age group, and cause of death were analyzed using chi-square or Fisher’s exact test, as appropriate. **Results:** Complete metopism was identified in 7 of 500 cases, corresponding to a prevalence of 1.4% (95% confidence interval: approximately 0.6–2.9%). No incomplete metopic sutures were observed. Metopism was slightly more frequent in females than males; however, no statistically significant association was found between metopism and sex, age group, or cause of death (*p* > 0.05). **Conclusions:** Persistent metopic suture is an uncommon but clinically and forensically relevant anatomical variant in adults. Its recognition during forensic autopsy is essential to avoid misinterpretation as a cranial fracture, particularly in trauma-related deaths, thereby preventing diagnostic and medico-legal errors.

## 1. Introduction

The metopic suture (median frontal suture) is located in the midline of the frontal bone at birth. Fusion of the suture typically begins between 9 months and 1 year of age and is expected to be completed between 6 and 8 years. This fusion progresses from the nasion to the bregma. In individuals where fusion does not occur, the suture remains as a persistent feature known as the metopic suture, or, when it extends continuously to the sagittal suture, it is referred to as metopism (persistent metopic suture). Metopism may present as either complete or partial [1,2,3,4]. Although mesenchymal cells play a central role in the embryological development and intramembranous ossification of cranial sutures, persistent metopic suture (metopism) is generally regarded as a normal anatomical variant rather than a manifestation of mesenchymal disease. Pathological mesenchymal conditions, such as craniosynostosis, are characterized by premature suture fusion and distinct clinical features, which are fundamentally different from the persistence of the metopic suture into adulthood [5]. The persistence of the metopic suture has been associated with abnormal cranial bone development, genetic factors, hereditary growth arrest, sexual and hormonal influences, atavism, cranial malformations, and hydrocephalus [4,6,7]. A thorough understanding of the morphology of the metopic suture and metopism is essential, as they may otherwise be misidentified as an extension of the sagittal suture or as cranial fractures. The metopic suture and metopism are nonmetric traits and are recorded as either present or absent. Therefore, their identification in the skull may serve as a parameter in forensic identification.

Most prevalence data on persistent metopic suture have been derived from dry skull collections or radiological series, particularly computed tomography-based assessments [8,9,10]. While valuable, these approaches may be affected by selection bias in curated skeletal collections, population representativeness, and variability related to imaging acquisition and interpretation. In contrast, autopsy-based evaluation allows direct macroscopic inspection of the frontal bone under standardized conditions and reflects real-world forensic practice, where persistent metopic sutures may be encountered in trauma-related deaths and misinterpreted as midline frontal fractures [11]. Therefore, autopsy-based studies provide a pragmatic anatomical reference that may reduce diagnostic and medico-legal errors by improving recognition of this normal variant [8,9,10,11].

The metopic suture and metopism have been frequently studied both in Turkey and worldwide, predominantly in anatomical laboratories or museums using dry skulls, or radiologically in living subjects [2,12,13,14,15]. However, they have only rarely been examined in forensic autopsy series [16] and some have been reported incidentally following autopsies of syndromic cases [17]. Previous studies conducted in Turkey have reported a metopism prevalence ranging from 4.6% to 9.3% [18], while globally, the prevalence of the metopic suture has been reported to range between 0% and 9.5% [4,19].

Although numerous studies have investigated the metopic suture, most have relied solely on dry skulls or radiologic methods [2,12,13,14,15]. Some metopic sutures have been detected incidentally during autopsies [17]. However, systematic studies based on autopsy series remain limited [16]. Both in Turkey and globally, there is a lack of cross-sectional studies assessing the frequency of metopic suture and metopism during autopsy.

Based on the hypothesis that autopsy-based macroscopic evaluation may provide more accurate and forensically relevant information on the prevalence and morphology of persistent metopic suture than studies relying solely on dry skull collections or radiological imaging, this study aimed to examine the presence of metopic suture and metopism in forensic autopsies and to contribute new data to the literature.

## 2. Materials and Methods

Following approval by the institutional ethics committee, this study was conducted at the Morgue Department of the İzmir Forensic Medicine Group Presidency. A total of 500 consecutive adult cadavers (aged 18 years and older) of both sexes, with intact cranial structures, were included. No selection was made based on gender, and all eligible cadavers were consecutively examined until the sample size was reached.

During the forensic autopsy, prior to craniotomy, the scalp was elevated in a standardized manner that did not interfere with the autopsy procedure or findings. A coronal incision was made extending from one auricle to the other across the vertex. The scalp was then reflected anteriorly and posteriorly to fully expose the frontal bone, including up to the nasion. The metopic suture was carefully inspected and recorded.

In each case, the presence of a metopic suture or metopism was documented. If present, it was classified as either complete (extending continuously from the nasion to the bregma) or incomplete (partially extending along the frontal bone midline). Photographic documentation was performed for all cases with visible metopic suture. Each case was recorded with demographic data (age, sex), and statistical evaluation was performed based on age groups and presence of the suture. In all cases with visible metopic sutures, photographic documentation was performed following a standardized protocol. Images were obtained after scalp reflection, with the frontal bone fully exposed, using a consistent camera distance, angle, and lighting conditions to ensure reproducibility and comparability across cases.

The study included adult cadavers aged 18 years or older with an intact cranial vault who underwent forensic autopsy at the İzmir Forensic Medicine Group Presidency. Cadavers were consecutively selected regardless of sex. Cases were excluded if the individual was younger than 18 years or if the cranial vault was damaged or deformed in a way that prevented evaluation of the frontal bone.

To ensure sufficient sample size, a power analysis was conducted. Based on institutional data, it was estimated that 1500 out of 2500 cadavers would meet the inclusion criteria, corresponding to a prevalence rate of 60%. At a 95% confidence level (z = 1.96) and α = 0.05 significance level, the minimum required sample size was calculated to be 370 cases. Therefore, a total of 500 cases were examined to exceed the minimum requirement and enhance the statistical power of the study [20].

As this study was based on forensic autopsy cases, the sample may overrepresent sudden, violent, or unnatural deaths. This selection characteristic may limit the generalizability of prevalence estimates to the general living population. However, the autopsy-based design is particularly relevant for the forensic context, where accurate recognition of anatomical variants such as persistent metopic suture is critical to avoid misinterpretation in trauma-related deaths.

All macroscopic evaluations were performed by a single experienced forensic physician. Formal inter-observer reliability analysis was not conducted, which represents a methodological limitation. However, the presence or absence of a persistent metopic suture is a clearly identifiable anatomical feature, and standardized inspection procedures were applied to minimize observer-related variability.

### Statistical Analysis

Statistical analyses were performed using SPSS version 27.0 (IBM Corp., Armonk, NY, USA). Categorical variables were summarized as frequencies and percentages, while continuous variables were expressed as median and range. Associations between the presence of metopic suture and sex, age group, and cause of death were analyzed using the chi-square test or Fisher’s exact test when expected cell counts were small. A *p*-value < 0.05 was considered statistically significant. The prevalence of metopism was calculated with corresponding 95% confidence intervals.

## 3. Results

Demographic Characteristics and Causes of Death Among Autopsy Cases are shown in Table 1. A total of 500 forensic autopsy cases were included in the study. The age of the cases ranged from 18 to 96 years, with a median age of 56 years. Regarding gender distribution, 387 were male (77.4%) and 113 were female (22.6%). When causes of death were analyzed, the most common was suspicious death, observed in 300 cases (60.0%). This was followed by traffic accidents in 50 cases (10.0%), gunshot wounds in 44 cases (8.8%), hanging in 33 cases (6.6%), falls in 25 cases (5.0%), cutting-piercing instrument wounds (CPIW) in 15 cases (3.0%), work accidents in 13 cases (2.6%), burns in 12 cases (2.4%), drowning in 6 cases (1.2%), assault in 1 case (0.2%), and electric shock in 1 case (0.2%) (Table 1).

**Figure 1:**
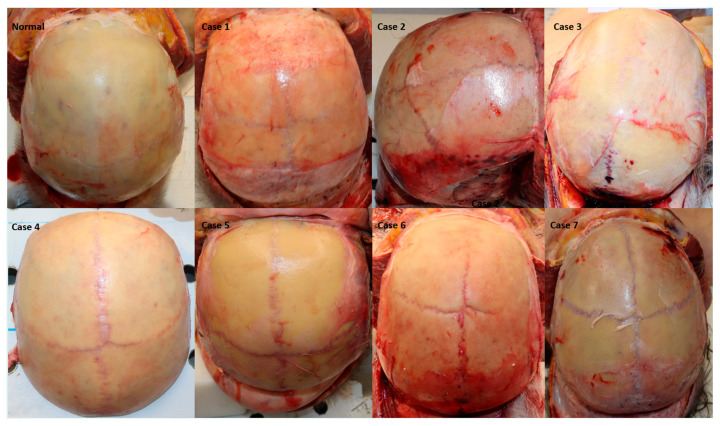
Forensic autopsy photographs demonstrating normal frontal bone and cases with complete persistent metopic suture. Images were obtained after standardized coronal scalp incision and reflection prior to craniotomy, allowing direct macroscopic inspection of the frontal bone.

**Figure 2:**
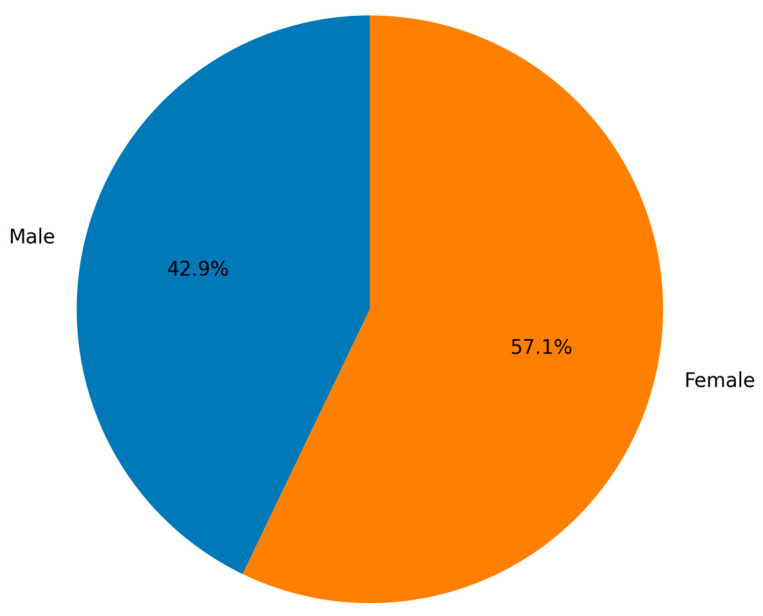
Gender distribution of adult autopsy cases with complete metopic suture (N = 7).

Frequency of Metopism Among Adult Autopsy Cases are shown in Table 2. Metopism was identified in 7 of 500 adult autopsy cases, corresponding to a prevalence of 1.4% (95% confidence interval: 0.6–2.9%). The median age of these cases was 67 years, ranging from 41 to 73 years. Of the 7 individuals with metopism, 3 (42.9%) were male and 4 (57.1%) were female. Regarding the cause of death, suspicious death was the most common, observed in 4 cases (57.1%), followed by fall-related deaths in 2 cases (28.6%), and one case (14.3%) attributed to acute myocardial infarction. No statistically significant association was found between the presence of metopism and cause of death (Fisher’s exact test, *p* > 0.05) (Table 2, Figure 1 and Figure 2).

**Figure 3:**
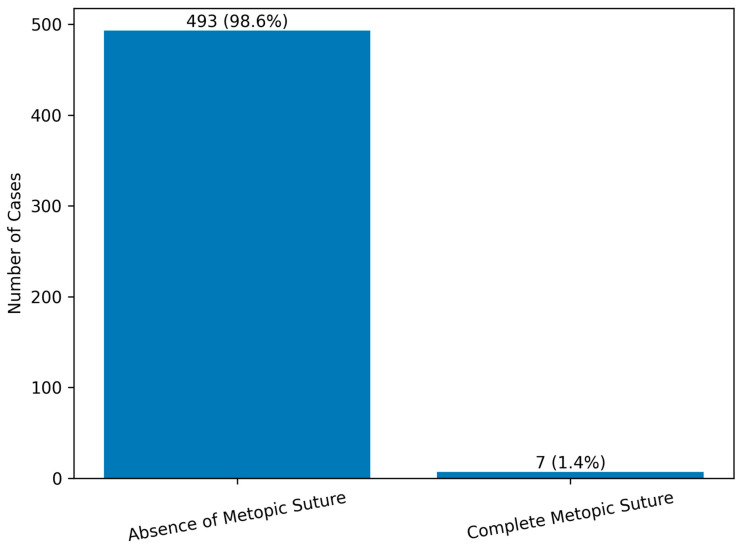
Distribution of metopic suture status among adult autopsy cases (N = 500).

Distribution of Metopic Suture by Gender is shown in Table 3. Among the 500 adult autopsy cases examined, the metopic suture was absent in 493 cases (98.6%), whereas complete metopism was identified in 7 cases (1.4%). When evaluated by gender, of those without metopic suture, 384 (76.8%) were male and 109 (21.8%) were female. The distribution of metopism did not differ significantly between sexes (female: 0.8% vs. male: 0.6%; Fisher’s exact test, *p* = 0.73). Among the 7 cases with complete metopism, 3 (0.6%) were male and 4 (0.8%) were female. Overall, the study population consisted of 387 males (77.4%) and 113 females (22.6%) (Table 3, Figure 3).

**Figure 4:**
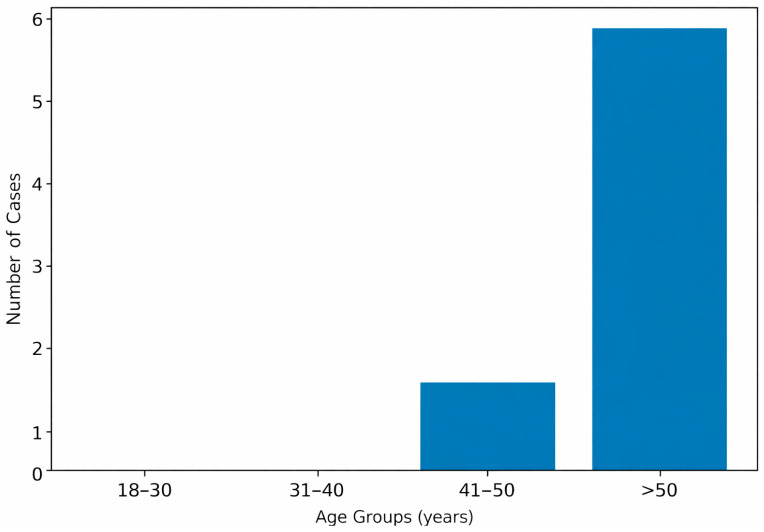
Age distribution of adult autopsy cases with complete metopic suture (N = 7).

Among the seven individuals with a complete metopic suture, the majority were aged over 50 years, accounting for 6 cases (85.7%). One case (14.3%) was identified in the 41–50 age group. No cases of complete metopic suture were observed in the 18–30 and 31–40 age groups. Although most cases with metopism were observed in individuals older than 50 years, the association between age group and presence of metopism was not statistically significant (Fisher’s exact test, *p* > 0.05) (Figure 4).

## 4. Discussion

In the present study, the incidence of persistent metopic suture (metopism) was identified as 1.4% among 500 adult autopsy cases. This prevalence was lower than that reported in most previous studies conducted in Turkey, where metopism rates ranged between 4.6% and 9.3%, based on dry skulls and radiological analyses [18,19]. The relatively lower incidence in our study may be attributed to methodological differences, as our data were obtained directly from forensic autopsy material rather than skeletal collections. This distinction adds considerable value to our findings, since autopsy-based assessments enable direct macroscopic evaluation under standardized conditions. Moreover, our study included a large and demographically diverse adult sample, enhancing its representativeness within the Turkish population.

The marked variability in the reported prevalence of persistent metopic suture across different populations may reflect underlying biological and genetic heterogeneity. Fusion of the metopic suture is a complex developmental process related to intramembranous ossification of the frontal bone, which is influenced by genetic regulation, cranial growth dynamics, and hormonal factors. Variations in these mechanisms may lead to delayed or incomplete fusion, allowing the suture to persist into adulthood as a normal anatomical variant rather than a pathological finding.

From a developmental perspective, persistent metopic suture represents a form of anatomical variation rather than disease. Its persistence without associated cranial deformity or neurological impairment suggests that, in many individuals, complete fusion is not biologically mandatory. This may explain why metopism can remain undetected throughout life and is often identified incidentally during radiological examinations or forensic autopsies.

Our findings revealed a complete metopic suture incidence of 1.4%, which is lower than many previous reports across different populations but falls within the broad range observed globally. While some studies have reported higher metopism rates—for example, Zanaty et al. observed a 42.9% metopic suture prevalence in Egyptian skulls [21], and Kamaşak et al. documented an 8.77% incidence of complete metopism in a Turkish sample [22]—others, such as Zdilla et al., reported much lower rates (1.8%) in the United States population [11]. Notably, Pakdeewong et al. found a 7.3% prevalence (including both complete and incomplete forms) in Thai specimens [23], while Nayakanati et al. reported 2.2% complete metopism in an Indian sample [24], which is more comparable to our results. These discrepancies may arise from differences in sample types (autopsy vs. dry skulls), detection methods (macroscopic vs. radiological), age distribution, and ethnic variation. Our study contributed to the literature as one of the few forensic autopsy-based assessments of metopism, providing directly observed macroscopic data rather than relying on skeletal or imaging analyses.

Compared with international studies, the prevalence of metopism in our autopsy-based series (1.4%) was relatively low. Zdilla et al. observed a slightly higher incidence of 1.8% in an American population [11], while Maskey et al. reported rates of 1.65% in Nepal and 2.88% in Korea, both exceeding our findings [5]. Similarly, researchers in Australia [25] and Nigeria [26] documented metopism rates of 4.8% and 1.04%, respectively. In contrast, much higher incidences were reported by Zanaty et al. in Egypt (8.2%) [21] and Kamaşak et al. in Turkey (8.77%) [22], both of which significantly exceeded the frequency observed in our study. These differences likely reflect variations in population genetics, sample types (e.g., autopsy vs. skeletal remains), and diagnostic criteria used across studies.

In our study, only complete metopic sutures were observed, with a prevalence of 1.4%, and no incomplete sutures were detected. This finding highlights a comparatively low incidence and a uniform presentation in terms of suture closure morphology. In contrast, Nayakanati et al. reported a much higher total incidence of 23.8% for metopic sutures, though only 2.2% represented complete metopism [24]. Similarly, Pakdeewong et al. observed 6.0% incomplete and 1.3% complete sutures in Thai skulls [23], demonstrating a broader variation in anatomical patterns across populations. These differences may stem from genetic, ethnic, or methodological factors, as well as sample type and study design.

In the present study, complete metopic sutures were slightly more common in females (0.8%) than in males (0.6%), although the difference was not statistically significant. This pattern is consistent with the findings of Şarbak et al., who observed a higher incidence of metopic sutures in females (13.2%) compared to males (3.2%) in an archaeological sample [27]. Conversely, studies by Nayakanati et al. and Gupta et al. either found no significant gender-based difference or reported a higher prevalence in males [24,28]. These discrepancies may be influenced by variations in sample size, population genetics, or study methodology.

In terms of age distribution, metopism was most frequently observed in individuals older than 50 years, accounting for 85.7% (6 out of 7 cases), while only one case (14.3%) was detected in the 41–50 age group. No cases were recorded in the younger age groups (18–30 and 31–40). These findings indicate that persistent metopic sutures may remain detectable primarily in older adults. This contrasts with some prior studies, such as that of Maskey et al., who reported visible metopic sutures in skulls up to the fifth decade of life [5], highlighting the potential for regional or methodological variability in age-related suture persistence. However, this observation should not be interpreted as age-dependent biological persistence. The apparent predominance of persistent metopic suture in older individuals may instead reflect sampling characteristics of forensic autopsy populations, increased detectability related to age-associated bone surface changes, and the limited number of cases. Therefore, no causal relationship between age or gestational differences and persistence of the metopic suture can be inferred.

Analysis by cause of death did not reveal any meaningful correlation with the presence of metopism. Among the seven identified cases, the most common cause of death was classified as suspicious (4 cases, 57.1%), followed by falls (2 cases, 28.6%), and acute myocardial infarction (1 case, 14.3%). No cases were associated with firearm injuries or hanging. These findings suggest that the presence of metopic suture was likely incidental and not linked to any specific cause of death. Nonetheless, the recognition of persistent metopic sutures during forensic autopsy remains critical, as they may be misinterpreted as skull fractures, particularly in trauma-related deaths, potentially leading to diagnostic errors [4,19].

Göksal et al. reported an incomplete metopic suture in a female individual from the Early Iron Age, suggesting a possible link between incomplete fusion and developmental delay or cranial pathology in adulthood [3]. However, in our study, no incomplete metopic sutures were identified, and all cases were classified as complete metopism. This discrepancy may reflect differences in the studied populations, time period, or methods of evaluation. Akbulut et al. identified a persistent metopic suture in 1 out of 12 adult male skulls (8.3%) and emphasized its morphological complexity and the importance of recognizing this variation during frontal craniotomies [2]. Our findings similarly underscore the necessity for both forensic and surgical professionals to be aware of metopism, as its presence—particularly when unrecognized—may lead to radiological or operative misinterpretation.

From a neurosurgical standpoint, awareness of persistent metopic suture is essential during frontal craniotomies and midline approaches. Failure to recognize this anatomical variant on preoperative imaging may lead to misinterpretation as a fracture line or influence surgical planning, particularly in trauma cases. Accurate identification can help avoid unnecessary extension of surgical exposure and reduce the risk of intraoperative complications.

In forensic anthropology and forensic medicine, persistent metopic suture holds particular importance. In trauma-related deaths, it may mimic a linear frontal fracture, potentially leading to erroneous conclusions regarding mechanism of injury. Moreover, the presence of metopic suture may influence frontal bone morphometric measurements used in sex estimation, underscoring the need for careful evaluation to prevent misclassification in medico-legal reporting.

One of the key strengths of our study was its autopsy-based design with a relatively large sample size. Unlike the majority of previous studies, which relied on dry skulls or radiological assessments, our investigation was based on direct macroscopic examination during forensic autopsies, allowing for accurate and standardized identification of metopic sutures under real-life conditions. This approach minimized the risk of misclassification due to postmortem damage or imaging limitations. To date, only a limited number of studies have assessed metopism using autopsy material, making our findings a valuable contribution to the anatomical and forensic literature [16].

Our findings provided autopsy-based evidence on the prevalence of complete metopic suture in the adult population, contributing new data to the anatomical understanding of metopism in forensic contexts. Although the incidence observed was relatively low (1.4%), its recognition remains important due to its potential to mimic cranial fractures or affect morphometric evaluations. As highlighted by Say and Okur, the presence of persistent metopic sutures may alter frontal bone measurements used for sex estimation in forensic anthropology, and failure to recognize this variation can lead to misinterpretation in medico-legal evaluations [29]. Our study reinforces the necessity for forensic experts to consider such anatomical variants during both external examination and radiological interpretation (Table 4).

### Limitations

This study has some limitations. First, the data were obtained from a single forensic institution, which may limit the generalizability of the findings to broader populations. Although the sample size was adequate, it may not have fully captured geographic, ethnic, or environmental variability. Additionally, since the study was based on forensic autopsy cases, there may have been an overrepresentation of unnatural or sudden deaths, potentially introducing selection bias.

Another important limitation is the absence of radiological correlation. As the evaluation relied solely on macroscopic inspection during forensic autopsy, no direct comparison with computed tomography or other imaging modalities could be performed; therefore, radiological–anatomical concordance of persistent metopic suture could not be assessed. Furthermore, inter-observer variability was not evaluated, as all assessments were conducted by a single experienced examiner. Although standardized inspection procedures were applied, the lack of formal inter-observer reliability analysis may introduce observer-related bias. These limitations should be considered when interpreting the results, and future studies combining autopsy-based evaluation with radiological imaging and multi-observer assessment are warranted.

## 5. Conclusions

In conclusion, this autopsy-based study provides population-specific forensic data on the occurrence of persistent metopic suture in adults and highlights its relevance as a normal anatomical variant encountered during forensic examinations. Despite its low frequency, recognition of complete metopism is essential to avoid misinterpretation as a frontal skull fracture, particularly in trauma-related autopsies. Overall, the findings underscore the value of autopsy-based macroscopic evaluation in forensic practice and support the need for future multicenter studies integrating radiological correlation to improve anatomical understanding and medico-legal interpretation.

## Figures and Tables

**Table 1:** Demographic Characteristics and Causes of Death Among Autopsy Cases (N = 500).

Variable	Cases (N = 500) N (%) or Median (Min–Max)
Age (Years)	56 (18–96)
Gender	
Male	387 (77.4%)
Female	113 (22.6%)
Cause of Death	
Suspicious Death	300 (60.0%)
Traffic Accident	50 (10.0%)
Gunshot Wound	44 (8.8%)
Hanging	33 (6.6%)
Fall	25 (5.0%)
Cutting-Piercing Instrument Wound (CPIW)	15 (3.0%)
Work Accident	13 (2.6%)
Burn	12 (2.4%)
Drowning	6 (1.2%)
Assault	1 (0.2%)
Electric Shock	1 (0.2%)

**Table 2:** Frequency of Metopism Among Adult Autopsy Cases (N = 500).

Variable n (%), Median (Min–Max)	Metopism Present (N = 7/500, 1.4%)
Age (Median, min–max)	67 (41–73)
Gender	
Male	3 (42.9%)
Female	4 (57.1%)
Cause of Death	
Suspicious Death	4 (57.1%)
Fall	2 (28.6%)
Acute Myocardial Infarction	1 (14.3%)

**Table 3:** Distribution of Metopic Suture by Gender.

Type of Suture	N (%)	Male, n (%)	Female, n (%)
Absence of Metopic Suture	493 (98.6%)	384 (76.8%)	109 (21.8%)
Complete	7 (1.4%)	3 (0.6%)	4 (0.8%)
Total	500 (100.0%)	387 (77.4%)	113 (22.6%)

**Table 4:** Comparison of the Present Study with Previous Literature Regarding the Incidence of Metopic Suture and Metopism.

Author	Incidence of Metopic Suture (%)	Incidence of Metopism (%)
Present Study	1.4 (100.0% complete)	1.4
Nayakanati et al. (2016, India) [24]	23.8 (Total: 2.2% complete + 21.6% incomplete)	2.2 (Complete Metopism)
Şarbak et al. (2017, Turkey) [27]	8.7 (Female:13.2%/Male: 3.2%)	Not specified
Zanaty A.W. (2017, Egypt) [21]	42.9	8.2
Zdilla MJ et al. (2018, USA) [11]	3.8	1.8
Vinchon et al. (2019, Thailand) [30]	5.1	3.4
Pakdeewong et al. (2019, Thailand) [23]	7.3 (6.0% incomplete + 1.3% complete)	1.3
Vidulatha et al. (2019, India) [31]	–	3.3
Vashist Y. et al. (2019, India) [32]	–	6.04
Andrade et al. (2019, Karnataka) [33]	44.29	1.43
Kamaşak et al. (2019, Turkey) [22]	8.77	8.77 (Complete)
Maskey et al. (2020, Nepal) [5]	27.27	1.65
Maskey et al. (2020, Korea) [5]	7.69	2.88
Tale et al. (2021, India) [34]	60	4
Chaisrisawadisuk et al. (2021, Australia) [25]	11.1 (4.8% complete + 6.3% partial)	4.8
Gupta et al. (2022, India) [28]	12.90	4.52
Edibamode et al. (2023, Nigeria) [26]	32.3	1.04
Say & Okur (2024, Turkey) [29]	– (only PMS reported)	4.9

## Data Availability

The data supporting the findings of this study are not publicly available due to legal and ethical restrictions, as they are derived from forensic autopsy records. However, anonymized data may be made available from the corresponding author upon reasonable request and with permission from the institutional ethics committee and relevant forensic authority.

## Abbreviations

MS	Metopic Suture
PMI	Postmortem Interval
CT	Computed Tomography
MRI	Magnetic Resonance Imaging
PMS	Persistent Metopic Suture
AMI	Acute Myocardial Infarction
CPIW	Cutting-Piercing Instrument Wound
MFGP	Forensic Medicine Group Presidency
SPSS	Statistical Package for the Social Sciences
SD	Standard Deviation
